# Usability of the Experience Sampling Method in Specialized Mental Health Care: Pilot Evaluation Study

**DOI:** 10.2196/48821

**Published:** 2023-11-21

**Authors:** Jeroen Dennis Merlijn Weermeijer, Martien Wampers, Lena de Thurah, Rafaël Bonnier, Maarten Piot, Peter Kuppens, Inez Myin-Germeys, Glenn Kiekens

**Affiliations:** 1 Center for Contextual Psychiatry KU Leuven Leuven Belgium; 2 Quantitative Psychology and Individual Differences KU Leuven Leuven Belgium; 3 Research Unit of Clinical Psychology KU Leuven Leuven Belgium; 4 Department of Medical and Clinical Psychology Tilburg University Tilburg Netherlands

**Keywords:** experience sampling, ecological momentary assessment, implementation, digital mental health, mobile phone

## Abstract

**Background:**

Mental health problems occur in interactions in daily life. Yet, it is challenging to bring contextual information into the therapy room. The experience sampling method (ESM) may facilitate this by assessing clients’ thoughts, feelings, symptoms, and behaviors as they are experienced in everyday life. However, the ESM is still primarily used in research settings, with little uptake in clinical practice. One aspect that may facilitate clinical implementation concerns the use of *ESM protocols*, which involves providing practitioners with ready-to-use ESM questionnaires, sampling schemes, visualizations, and training.

**Objective:**

This pilot study’s objective was to evaluate the usability of an ESM protocol for using the ESM in a specialized mental health care setting.

**Methods:**

We created the ESM protocol using the m-Path software platform and tested its usability in clinical practice. The ESM protocol consists of a dashboard for practitioners (ie, including the setup of the template and data visualizations) and an app for clients (ie, for completing the ESM questionnaires). A total of 8 practitioners and 17 clients used the ESM in practice between December 1, 2020, and July 31, 2021. Usability was assessed using questionnaires, ESM compliance rates, and semistructured interviews.

**Results:**

The usability was overall rated reasonable to good by practitioners (mean scores of usability items ranging from 5.33, SD 0.91, to 6.06, SD 0.73, on a scale ranging from 1 to 7). However, practitioners expressed difficulty in personalizing the template and reported insufficient guidelines on how to use the ESM in clinical practice. On average, clients completed 55% (SD 25%) of the ESM questionnaires. They rated the usability as reasonable to good, but their scores were slightly lower and more variable than those of the practitioners (mean scores of usability items ranging from 4.18, SD 1.70, to 5.94, SD 1.50 on a scale ranging from 1 to 7). Clients also voiced several concerns over the piloted ESM template, with some indicating no interest in the continued use of the ESM.

**Conclusions:**

The findings suggest that using an ESM protocol may facilitate the implementation of the ESM as a mobile health assessment tool in psychiatry. However, additional adaptions should be made before further implementation. Adaptions include providing training on personalizing questionnaires, adding additional sampling scheme formats as well as an open-text field, and creating a dynamic data visualization interface. Future studies should also identify factors determining the suitability of the ESM for specific treatment goals among different client populations.

## Introduction

### Background

Mental problems are inextricably linked to daily life, meaning they do not exist in a vacuum but are influenced by our everyday activities, environments, and social interactions; for example, in the case of individuals diagnosed with schizophrenia spectrum disorder, work-related activities may decrease hallucinatory intensity over time [1]. By contrast, paranoid ideation may develop in response to the social environment [2] or stress [3]. This indicates that mental health problems can best be understood when they are investigated in the context in which they occur: a client’s daily life. Unfortunately, however, practitioners are confined to the brick-and-mortar walls of their therapy room and must rely on the clients’ ability to retrospectively report their feelings, thoughts, and behaviors. This approach may provide only partial insights owing to selective or incomplete recall biases [4] because clients cannot be expected to accurately remember and share all relevant daily life experiences and associated emotions or symptoms. Therefore, complementary tools that can aid clients in reliably sharing their daily life experiences with their practitioners could be valuable for clinical practice; for instance, such tools may benefit therapy by increasing insight into the contextual variability of mental health problems as they are observed across different contexts in people’s daily lives. Although this was not feasible for many decades owing to practical restrictions, smartphone technologies can now enable practitioners and clients with new digital tools to bring this highly relevant information into the therapy room.

The experience sampling method (ESM) [5-7] is a structured diary technique that involves using smartphone apps to assess thoughts, feelings, symptoms, and behaviors in clients’ daily environments. Individuals are prompted to complete a brief questionnaire multiple times a day for several consecutive days. The questionnaire is completed *in the moment* and typically contains questions about people’s momentary thoughts, feelings, symptoms, behaviors, and situational circumstances (eg, “Who are you with?” “Where are you?”). Individuals may also be asked to rate sleep quality in the morning or evaluate the day in the evening.

The ESM may have value for clinical practice for several reasons. First, because ESM questionnaires facilitate real-time assessment, the risk of recall bias is reduced compared with traditional assessment methods such as clinical interviews or retrospective questionnaires [8,9]. Second, through self-monitoring by clients multiple times per day, the ESM may increase self-insight and emotional self-awareness [10]. In a similar vein, the ESM may help identify protective factors in the environment that may facilitate resilience, such as social networks that can provide support in moments of high distress [11]. Third and last, the ESM may help practitioners to explore and develop hypotheses about the underlying factors for mental health problems. Similarly, the ESM could be used to evaluate whether the provided treatment has the desired effects on clients’ everyday lives (eg, improved mood and reduced symptoms) and, if required, to make changes to the treatment plan collaboratively with clients [6].

Emerging evidence shows that mental health practitioners and clients recognize the ESM’s potential advantages and are generally interested in using it in clinical practice [12,13], although some findings also suggest that practitioners may not adopt the ESM more readily than traditional assessment tools [14]. However, clients might be more favorable toward the actual use of the ESM in clinical practice than practitioners [15]; for instance, meta-analyses found good compliance rates for ESM questionnaires (>75% on average) among clinical samples [16]. However, implementing the ESM in mental health care has proven challenging because implementation attempts so far have failed to promote continued or far-reaching use [15,17-19]. One possible reason for this may be that the ESM software in use was originally designed for research purposes without the involvement of input from practitioners and clients. This can lead to usability problems when implementing the software in clinical practice; for instance, previous studies have highlighted that practitioners found the ESM software to be too time intensive and not intuitive, making it challenging to effectively use the ESM data for clinical purposes [19]. These findings suggest that an in-depth investigation into end-user software requirements may be necessary before it becomes feasible to use the ESM as a clinical tool in mental health care.

In response to the lack of end-user perspectives on clinical ESM software requirements, we recently conducted a qualitative focus group study with mental health care practitioners to understand better how they wanted to use the ESM and which elements this would require [20]. One important finding that emerged was the need for *ESM templates* detailing the ESM questionnaire content and sampling schedule so that practitioners do not need to develop this for each client. At the same time, practitioners stressed that personalization should still be possible, such as creating new ESM items or a schedule tailored to a client’s needs. Furthermore, they recommended using intuitive data visualizations such as line graphs depicting mood variability over time or pie charts displaying the frequency of contact with others (eg, family vs friends). Finally, practitioners expressed a need for training and guidelines on using ESM templates, personalization, and data visualizations. Similar findings were recently found in a survey among 89 practitioners [13].

Although these initial findings offer some insights into the requirements of software for clinical ESM applications (eg, templates and intuitive data visualization) and the implementation strategies more generally (eg, need for user training), it is important to consider that the practitioners had no prior experience with using the ESM in clinical practice. Therefore, whether accommodating these identified needs will make the ESM effectively usable in routine mental health care (ie, intended vs actual use) remains to be investigated. Hence, a practical next step is to create an ESM *protocol* based on these recommendations and evaluate end-user experiences by piloting the ESM protocol with practitioners and clients before wider dissemination [21]. Such a multitiered approach provides meaningful information about the barriers and facilitators experienced, as well as requirements for future ESM implementation efforts. To this end, we designed a protocol called Implementing Personalized Real-Time Monitoring in Everyday Life (IMPROVE), in which the ESM templates containing the scheduled ESM questionnaire content and sampling schedules can be tailored to the needs of clients who self-monitor for a week using the ESM app. The collected information is automatically visualized through intuitive visualizations by the practitioner on their dashboard. The dashboard and app were created using the m-Path software platform [22], and the user training and guidelines were based on previous work [12,20].

### Objectives

This pilot study’s objective was to evaluate the usability of IMPROVE. Specifically, we were interested in whether the IMPROVE dashboard and app were considered acceptable and easy to use as well as whether practitioners and clients were satisfied with the user interface design. For clients, and although the thresholds are somewhat arbitrary [23], we also wanted to investigate whether they would complete at least one-third of the assessments (20/60, 33%). In addition, we were interested in practitioners’ and clients’ perspectives on the ESM items and sampling scheme, options for personalization, the design of the dashboard and data visualizations, and the training material. To accomplish these objectives, we used a mixed methods approach for which the analysis plan was preregistered [24].

## Methods

### Participants

The study targeted mental health practitioners working at KU Leuven’s University Psychiatric Centre in Leuven in the Flanders region of Belgium. Practitioners were recruited exclusively via email owing to COVID-19 restrictions at the time of recruitment. To be eligible for participation, certified practitioners had to be working with clients experiencing mental health problems and have good Dutch language proficiency. Practitioners recruited clients into the study based on their assessment of whether IMPROVE would be helpful for the particular client. Clients were required to be aged ≥18 years, to be receiving residential or ambulant mental health care, to have good Dutch language proficiency, and to own a smartphone with at least third-generation (3G) network coverage. We used these broad selection criteria to ensure a comprehensive and realistic assessment of the usability of IMPROVE across clients presenting with different mental health problems and at different treatment stages.

### Procedure

Practitioners first provided written informed consent and completed an enrollment questionnaire assessing sociodemographic information. Subsequently, they were provided with a manual on how to use the ESM. The manual included instructions on creating an account on the ESM dashboard, setting up their account to access the ESM questionnaire content and sampling schedule, enrolling a client, adjusting or creating questionnaires and sampling schemes, and visualizing data paired with interpretation examples. In addition, practitioners could join a 1-hour web-based training session with a research team member, which covered the same topics as the manual. Once practitioners were familiar with using the ESM dashboard, we requested them to use it with several clients in their clinical practice.

The practitioners informed clients about the study, and if a client showed interest, they were asked to read and sign the informed consent form (because the research team was not allowed access to the hospital owing to the COVID-19 restrictions). After providing informed consent, clients completed enrollment questionnaires that assessed demographic and clinical variables. Afterward, clients installed the m-Path smartphone app, which was used to trigger the ESM questionnaires. The practitioners could personalize the ESM questionnaires for the client using the m-Path dashboard. After 1 week of using the ESM, practitioners and clients were requested to discuss the visualizations of the client’s data during the subsequent clinical session using the dashboard. To assess any operational difficulties or software bugs, we telephoned practitioners biweekly for routine check-ins during which they could report technical problems or difficulties with using the software.

At the end of the implementation period, clients and practitioners were provided with questionnaires assessing the usability of the IMPROVE software and invited to participate in a semistructured interview. The interview allowed us to capture more rich information about the experiences of practitioners and clients regarding the use of the ESM. The pilot study took place between December 1, 2020, and July 31, 2021, and was in accordance with the ethical principles of the American Psychological Association [25].

### Materials

#### IMPROVE Dashboard, App, and Training Material

The IMPROVE protocol used m-Path’s ESM software platform [22], which consists of an app to deliver the ESM questionnaires and a dashboard on which ESM questionnaires can be created and data visualized. Specifically, making use of the custom-made *ESM template* feature (developed by m-Path for this project), we provided practitioners with an ESM template that contained a ready-to-use ESM questionnaire that followed a predefined sampling scheme (ie, 10 ESM assessments/d for 6 d as well as morning and evening assessments). The ESM monitoring period started 1 day after a client had downloaded the ESM app and registered with their practitioner. In addition, the template could be personalized because practitioners could add a maximum of 3 ESM questions from a library, adjust existing multiple-choice options to include more answer options (eg, “Who are you with?”), and modify the sampling schedule to fit sleep-wake patterns. The ESM content consisted of a morning questionnaire assessing sleep quality and motivation to start the day, an evening questionnaire evaluating people’s day overall, and an ESM questionnaire containing questions on mood, location, and activity (Textbox 1; for additional content, please refer to our preregistration page [24]). Although the morning and evening questionnaires were assessed once daily, clients received the ESM questionnaires 10 times per day for 6 consecutive days between 7:30 AM and 10:30 PM by default [26]. Five minutes after clients had received a beeping alert but had not responded, a reminder was sent to them to fill out the ESM questionnaire. The app was synchronized with the dashboard, and responses were automatically visualized on the dashboard. Implementation strategies included making use of a user manual with guidelines, including video links explaining how to use the dashboard and one-on-one web-based training sessions.

**Content of the experience sampling method (ESM) template (unless specified otherwise, items were answered on a scale ranging from 1=not at all to 7=very much).**

**Morning questionnaire**

1. At what time did you go to sleep yesterday? (Numeric input)
2. How long did it take you to fall asleep? (Less than 10 min or less than 30 min or less than 1 h or more than 1 h)
3. How many times did you wake up last night? (I slept uninterrupted or 1 time or 2 times or more than 2 times)
4. I slept well.
5. At what time did you wake up this morning? (Numeric input)
6. How long did you lie awake before you got up? (Less than 10 min or less than 30 min or less than 1 h or more than 1 h)
7. I am excited to start the day.
8. How many hours did you sleep last night? (I could not sleep or between 1 and 2 h or...or between 9 and 10 h or more than 10 h)

**ESM questionnaire**

1. I feel lonely.
2. I feel anxious.
3. I feel stressed.
4. I feel sad.
5. I feel insecure.
6. I feel satisfied.
7. I feel cheerful.
8. I feel excited.
9. I feel relaxed.
10. What am I doing? (Leisure–active [eg, playing games and sports] or leisure–passive [eg, watching television and reading] or school or work or everyday chores [cooking, cleaning, and shopping] or traveling or eating or drinking o social contact or something else or nothing)
11. I like doing this.
12. I’d rather do something else.
13. Where am I? (At home or at a friend of family member’s home or at work or public transport or somewhere else outside or somewhere else inside)
14. Who am I with? (Nobody or family or friends or colleagues or other familiar people or unfamiliar people)
15. Conditions
  - (Alone) I like being alone
  - (With others) I would rather be with others
  - (With others) I like this company
  - (With others) I would rather be alone
16. (Optional example) To what extent have you experienced burden or discomfort since the last beep because of compulsions?

**Evening questionnaire**

1. I thought this was a normal day.
2. I thought this was a nice day.
3. What was the most NEGATIVE event of the day for you? (Open text)
4. How unpleasant was this event?
5. How enjoyable was this event?
6. Completing the questionnaires on this app has influenced my mood throughout the day.

#### ESM Data Visualizations

**Figure 1:**
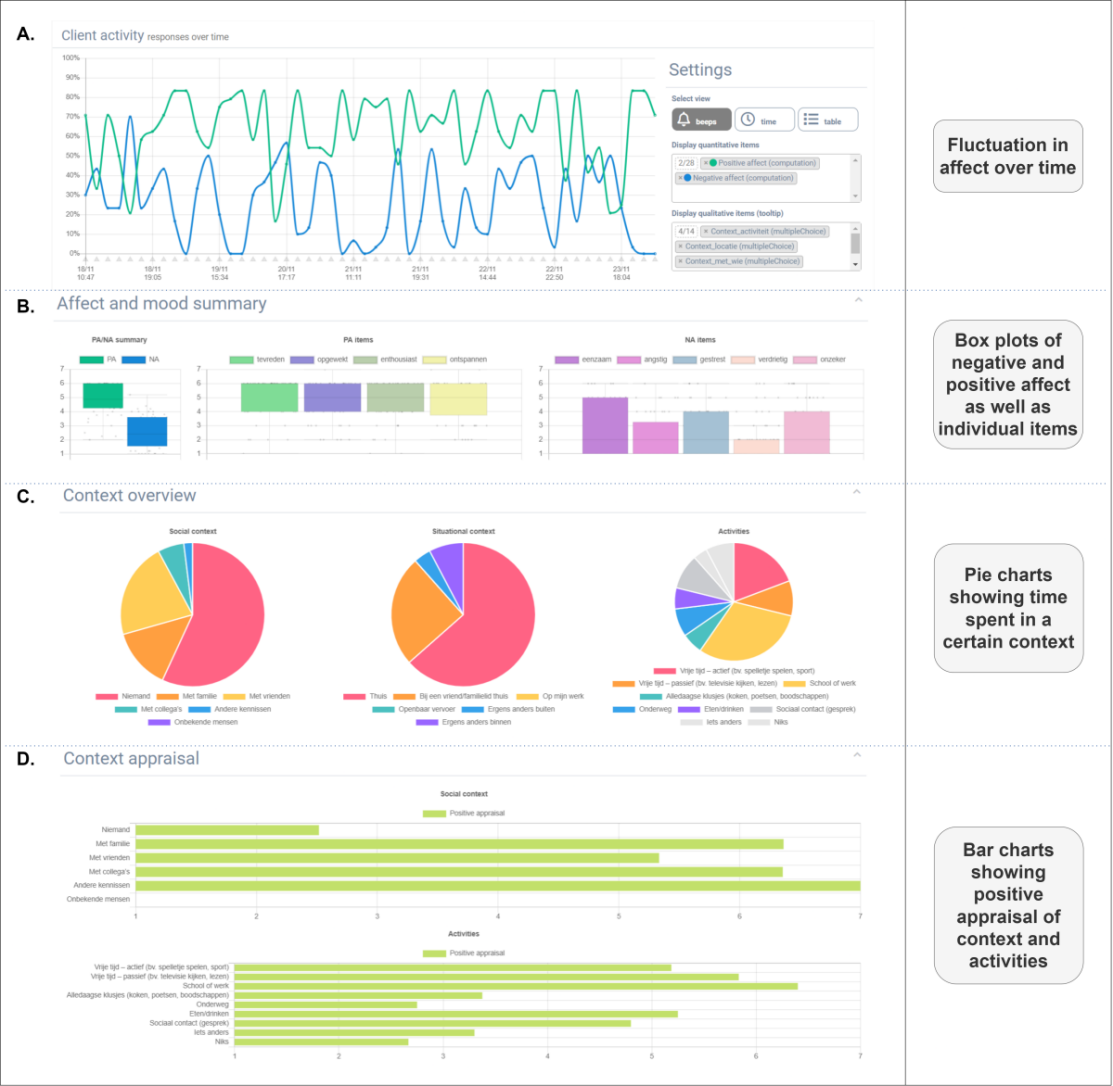
Examples of visualizations used in the Implementing Personalized Real-Time Monitoring in Everyday Life protocol.

The data collected with the ESM app were displayed on the web-based dashboard using a series of custom-made visualizations. These visualizations were based on end-user input on existing visualization methods for ESM data [20], including information on general psychological functioning (ie, box plots of positive and negative emotions) and contextual information across the ESM period (eg, pie charts expressing the distribution of time alone vs with others face-to-face or on the web and activities in daily life), fluctuations over time (eg, time series graphs of negative affect), and qualitative text tables with descriptions of the most pleasant event of the day. For the time series plot, it was also possible to zoom in on a single measurement point and relate the data point of interest to contextual information (eg, with who someone was, where they were, and what they were doing). Affect or symptom items could also be made conditional on contexts or activities to provide insight into the contextual determinants of mental health problems (eg, symptom frequency at home vs symptom frequency at work). Figure 1 provides examples of visualizations used in IMPROVE.

### Quantitative Measures

#### Sociodemographic Information

At enrollment, clients and practitioners provided age and sex information. In addition, practitioners provided information on their profession (eg, psychiatrist or clinical psychologist).

#### Compliance

Compliance with the ESM protocol was assessed as the percentage of completed versus scheduled ESM assessments.

#### Questionnaires on Usability

To evaluate the usability of the ESM app and dashboard, we used an adapted version of the mHealth App Usability Questionnaire (MAUQ) [27]. The MAUQ assesses different usability elements, such as whether clients and practitioners found the app and dashboard easy to use or were satisfied with the user interface (eg, “I found it easy to learn to use the dashboard”). Items were rated using a 7-point Likert scale ranging from 1 to 7, with higher scores indicating higher usability of the app for clients and the dashboard for practitioners. Given that the questionnaire’s statements assess different meaningful usability aspects, we interpreted individual items and did not calculate a composite score. For practitioners, we differentiated between the usability of the dashboard during a clinical session and overall usability. We made this decision because practitioners could use IMPROVE several times with multiple clients, whereas clients only used IMPROVE with 1 practitioner.

### Qualitative Measures

Practitioners and clients were invited to participate in a semistructured interview after using IMPROVE. The interview assessed in-depth experiences of using the ESM in practice, the provided training material, the ESM items and sampling scheme, personalization options, data visualization, and suggestions for improvement. Interview guides were developed and divided into thematic sections (eg, expectations regarding ESM implementation in mental health care, technical feasibility, and the ESM template), with each section starting with a short introduction to the topic. Sections were composed of broad questions followed by more specific prompt questions. The interviewers were allowed to make minor changes to the phrasing of the questions to make them sound more natural but were instructed not to change the content and meaning of the questions.

### Data Analysis

#### Quantitative Data Analysis

Concerning compliance, we evaluated whether clients provided sufficient data for making reliable inferences of automated data analysis at the intraindividual level [28]. Although the thresholds are somewhat arbitrary [23], we assessed whether clients could complete a minimum of 33% (20/60) of all provided questionnaires. For the adapted MAUQ, the scores of individual items that assessed usability were visualized and interpreted using heat maps (Multimedia Appendices 1-3).

#### Qualitative Data Analysis

The audio recordings of the interviews were transcribed and analyzed based on inductive data-driven thematic analysis [29]. This involves several consecutive steps. First, the lead author familiarized himself with the data by relistening to the audio recordings. Second, the transcripts were reread and divided into meaningful text segments. Third and last, these segments were labeled with short summarizing and comprehensible sentences (ie, open coding approach). Afterward, the labeled segments were grouped into subthemes. Subsequently, these subthemes were grouped into overarching themes. How the different segments were grouped into subthemes and overarching themes was refined through collaboration with coauthors. In addition, we included a second coder to evaluate the reliability of the results. The second coder was provided with 10% of the unlabeled segments of the lead author (randomly selected) and was asked to label them and group them into subthemes and overarching themes. Afterward, we compared the coding and evaluated the labeling and grouping agreements to assess interrater reliability. Cohen κ was subsequently calculated as the percentage of agreement.

#### Dropouts and Missing Data

Given that dropouts may indicate poor usability, the number of dropouts is reported for both clients and practitioners.

### Ethical Considerations

Practitioners and clients were requested to provide written informed consent, and the study was approved by the medical ethics committee of KU Leuven (Leuven, Belgium; S64244). To align with real-world conditions, no remuneration was provided to practitioners and clients for participating in this study. Participants were given aliases on all transcriptions of recordings. Personal information obtained from interviews was not linked to individual identities. Any written or printed documents containing information on the identity of participants, such as informed consent forms, were stored in a locked archiving room. The data presented in this paper do not contain real names or any information that reveals the identities of the participants.

## Results

### Participants

We invited 142 practitioners to participate in the study, of whom 12 (8.5%) initially agreed to participate. Of the 12 practitioners, 11 (92%) attended the optional web-based training session, and 8 (67%) used the IMPROVE protocol with clients in therapy. The practitioners who did not use the IMPROVE protocol (4/12, 33%) mentioned the excessive burden of trying out novel instruments amid the COVID-19 pandemic and clients not showing up for scheduled appointments as reasons for not using IMPROVE.

The practitioners invited 29 clients to participate (mean 2.42, SD 1.80 clients/practitioner), of whom 24 (83%) agreed. Of the 24 participants, 17 (71%) completed the study. Dropouts occurred at various points: of the 24 clients, 1 (4%) decided to quit during baseline data collection, and 6 (25%) ended the ESM week but did not attend the clinical feedback session or did not complete the usability questionnaires. Table 1 provides the demographic characteristics of the practitioners and clients who participated in the pilot study.

**Table 1:** Demographic characteristics of clients and practitioners.

					Users (practitioners: n=8, clients: n=17)			Dropouts or nonusers (practitioners: n=4, clients: n=12)
**Practitioners**								
	Age (years), mean (SD)			45.57 (6.11)			43.50 (17.50)	
	Sex (female), n (%)			7 (88)			1 (25)	
	**Profession, n (%)**							
		Clinical psychologist	5 (63)			1 (25)		
		Mental health nurse	2 (25)			N/A^a^		
		Neuroscientist	1 (13)			N/A		
		Psychiatrist	N/A			3 (75)		
**Clients**								
	Age (years), mean (SD)			34.93 (11.27)			36.67 (13.47)	
	Sex (female), n (%)			11 (65)			5 (42)	

^a^N/A: not applicable.

### Quantitative Analysis

#### Practitioner Ratings of the Usability of the Dashboard

##### Usability of the Dashboard During a Clinical Session

Regarding the usability of the ESM dashboard during a clinical session, mean responses to individual items ranged from 5.33 (SD 0.91) to 6.06 (SD 0.73). Practitioners reported the lowest agreement to the statement “When I made a mistake, I could correct it easily and quickly,” whereas the highest agreement was reached for the statement “I felt comfortable talking to my client about the data that were visualized on the dashboard.” When inspecting the heat map of responses (Multimedia Appendix 1), we observe saturation around *6=agree* for all statements, apart from responses to the statement “The information on the dashboard was well organized, I could easily find what I needed for this session,” for which responses were saturated around *5=somewhat agree*.

##### Overall Usability of the Dashboard

Regarding the overall usability of the ESM dashboard, mean responses to individual items ranged from 4.00 (SD 1.91) to 6.14 (SD 0.69). The lowest and highest levels of agreement were reached for the statements “It was easy for me to learn to use the dashboard” and “I would use the dashboard again,” respectively. In the plotted heat map (Multimedia Appendix 2), we observe saturation for 8 (73%) of the 11 usability statements labeled *5*=*somewhat agree*, *6=agree*, and *7=strongly agree*. The usability statements that deviate from this pattern (3/8, 38%) were “It was easy for me to learn to use the dashboard,” “It was easy for me to create questionnaire content,” and “It was easy for me to schedule or reschedule questionnaires.”

#### Client Ratings of the Usability of the ESM App

Mean responses to the app usability statements varied between 4.18 (SD 1.70) and 5.94 (SD 1.50), with the lowest and highest levels of agreement reached for the statements “The app had all features and capabilities I expected of it” and “I felt comfortable talking to my clinician about the data collected with the app,” respectively*.* For 9 (75%) of the 12 statements, the largest percentage of responses consisted of *5=somewhat agree*, *6=agree,* and *7=strongly agree*. The remaining statements (3/12, 25%), which mainly concerned the design and user interface of the app, were characterized by a large degree of variation; for example, 35% (6/17) of the clients answered *agree* to the statement “From the start, I found the app easy to use,” whereas 24% (4/17) answered *disagree* (Multimedia Appendix 3).

#### Compliance With Scheduled ESM Assessments

On average, clients completed 55% (SD 25%; range 18%-93%) of the ESM questionnaires (excluding morning and evening questionnaires). However, 5 clients did not reach the predefined threshold of 33% (range 18%-31%), indicating limited feasibility for reliable inference. In addition, one client stated unwillingness to share their ESM data with the research team.

### Qualitative Data Analysis

#### Overview

**Figure 2:**
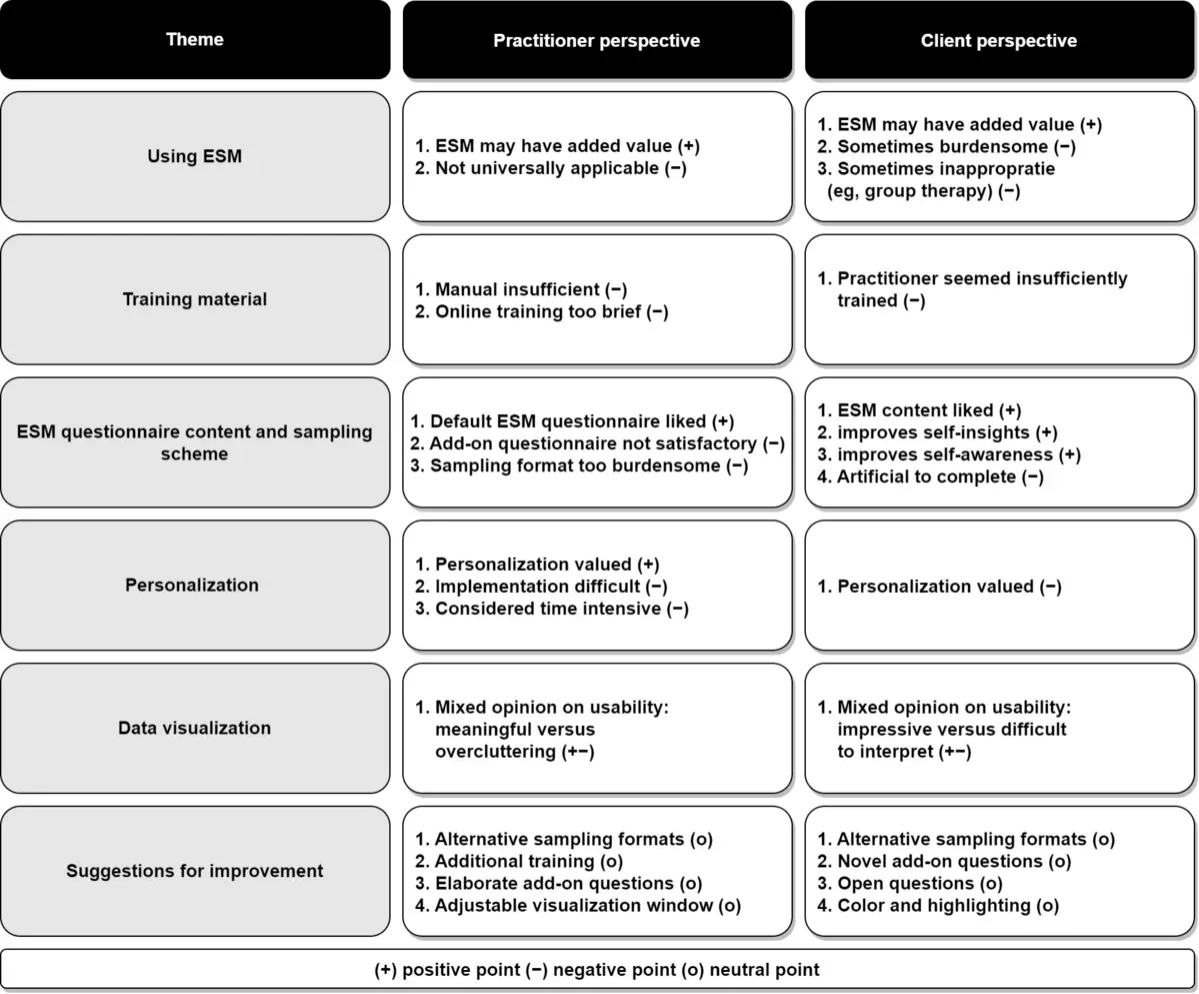
Summary of the results from thematic analysis on usability. ESM: experience sampling method.

The overarching themes were using the ESM in clinical practice, the training material, ESM content, personalization, data visualization, and suggestions for improvements. For each of these themes, subthemes were identified. Figure 2 summarizes the results, and Multimedia Appendix 4 includes example quotes.

#### Using the ESM in Clinical Practice

Practitioners seemed engaged with the software, with multiple practitioners expressing a desire for continued use (quote 1 in Multimedia Appendix 4). However, practitioners mentioned that several clients declined to participate because they considered a week of using the ESM with the default sampling scheme too burdensome or they had no smartphone or internet access (quote 2 in Multimedia Appendix 4). Some of the clients who participated also voiced these concerns and indicated that the ESM sampling scheme was too burdensome or that the noise or vibration from a notification was disturbing in some situations, such as during a group therapy session or relaxation exercise (quotes 3 and 4 in Multimedia Appendix 4). In addition, 1 client reported that it felt artificial to reduce emotions and cognitions to ratings on a scale and felt that they were expected to show variation in their responses (quote 5 in Multimedia Appendix 4).

#### Training Material

The training material we provided was generally considered useful but not practical; for example, 1 practitioner indicated difficulties after the web-based training on a tablet computer, indicating a preference for group training with colleagues present (quote 6 in Multimedia Appendix 4). Another practitioner mentioned that the manual we provided would take too much time to go through and instead relied on learning with the help of a colleague also enrolled in the study (quote 7 in Multimedia Appendix 4). Similarly, 1 client said they felt confused about the necessity of the repetitive nature of the ESM (quote 8 in Multimedia Appendix 4), indicating insufficient briefing by the practitioner before the implementation of the ESM.

#### ESM Items and Sampling Scheme

Mixed opinions existed on the usability of the predefined ESM questionnaire. Practitioners found the default ESM items (eg, questions on emotions, context, and activities) relevant but were skeptical of some of the add-on items, stating that they were unsatisfied with the phrasing (eg, a focus on *burden* in the assessment of obsessive-compulsive disorder symptoms; quote 9 in Multimedia Appendix 4). Similarly, some practitioners found a single week of the ESM too brief to detect meaningful changes and indicated that brief periods of intensive ESM might be more appropriate during the initial stages of therapy (quote 10 in Multimedia Appendix 4). Similar to practitioners’ views, clients generally found the default ESM content relevant and mentioned that it increased insight as ESM made them more aware of their feelings and behaviors (quote 11 in Multimedia Appendix 4).

#### Personalization of the ESM Questionnaire

Practitioners generally found the options for personalization valuable (quotes 12 and 13 in Multimedia Appendix 4). However, most practitioners mentioned that they rarely personalized the ESM questionnaire, which was perceived as too complex and time consuming (quote 14 in Multimedia Appendix 4); for example, 1 practitioner stated that they might need assistance in customizing the ESM questionnaire because they were “not good with technology” (quote 15 in Multimedia Appendix 4).

#### Data Visualization of Collected ESM Data

Although some practitioners liked the visualizations and indicated that they helped them and their clients to concretize the contextual nature of mental health problems (quotes 16 and 17 in Multimedia Appendix 4), others found it initially overwhelming and indicated the need for practice to make sense of the different visualizations (quote 18 in Multimedia Appendix 4). Similarly, although some clients considered the data visualizations informative (quote 19 in Multimedia Appendix 4), others found it overwhelming and challenging to know what was relevant (quote 20 in Multimedia Appendix 4). To illustrate, 1 client reported that they would not use the ESM without a practitioner by their side to help them understand how to interpret and give meaning to the results (quote 21 in Multimedia Appendix 4).

#### Suggestions for Improvement

Practitioners and clients made suggestions for improvement related to different elements of the ESM protocol. First, regarding the ESM template, practitioners and clients indicated the need for alternative sampling formats compared with a single observation period with 10 beeping alerts daily; for instance, a practitioner suggested using the ESM at different periods in the therapy to evaluate progress (quote 22 in Multimedia Appendix 4). Relatedly, clients indicated sampling 1 week a month with fewer beeping alerts to make it less burdensome (quote 23 in Multimedia Appendix 4). Second, practitioners reported that additional training is necessary, which could include more case descriptions and mock sessions (quote 24 in Multimedia Appendix 4). Third, concerning the ESM content, some clients found that the ESM questionnaires were too generic and suggested using open questions and responses (quotes 25 and 26 in Multimedia Appendix 4). Relatedly, additional add-on questions were requested by practitioners and clients to monitor a broad range of experiences related to substance abuse, obsessive-compulsive disorder, stress, and physical health. Fourth, regarding data visualizations, some practitioners expressed the desire for adjustable visualizations, such as making it possible to annotate and adjust visualizations (quote 27 in Multimedia Appendix 4). Fifth and last, clients said that using more color or highlighting important parts of a question might be worthwhile to make filling out the ESM questionnaire less monotonous and more time efficient (quotes 28 and 29 in Multimedia Appendix 4).

## Discussion

### Principal Findings

Despite the potential benefits of the ESM in terms of making clients more actively involved and better matching treatments to their needs [6,10,11], it is still primarily used in research settings with little uptake in clinical practice. In this study, we piloted the usability of an ESM protocol for employing ESM in a specialized mental health care setting for clients in need of psychiatric care. This consisted of an ESM template with ready-to-use ESM questionnaires, sampling schemes, and visualizations, as well as add-on materials. The ESM template was implemented through a dashboard for practitioners (ie, including the setup of the template and data visualizations) and an app for clients (ie, for completing the ESM questionnaires). Our results indicate that working with ESM templates can facilitate usability but suggest that a single generic template will be insufficient to capture clients’ needs and address clinical goals in practice.

Although clients were somewhat less favorable than practitioners, we observed that the technical usability of the piloted software was considered sufficient by both practitioners and clients. However, 2 findings warrant further discussion. First, although practitioners found the template easy to use and expressed a willingness to use it again, they also encountered difficulties when attempting to use more advanced features, such as personalization, and when displaying and interpreting data visualizations; for instance, some practitioners reported feeling overwhelmed when presented with multiple data visualizations, not knowing where to focus their attention. Interestingly, this feeling of being overwhelmed was also observed in some clients who found it challenging to understand what was relevant. One possible solution to address this issue could involve presenting a limited number of visualizations per web page or implementing a dynamic visualization interface that allows users to select or deselect specific visualizations for viewing. Second, from the clients’ perspective, several individuals mentioned that the tool lacked expected features, with the inability to provide additional momentary information in an open-text field being the most noteworthy deficiency. Specifically, the desire for open-text fields may indicate that clients found it challenging to recall qualitative daily life experiences solely based on quantitative daily life data; for instance, when a practitioner wants to discuss an observed peak in mood (whether positive or negative), it might be difficult for the client to recall the specific contextual details of that moment during a therapy session, even when provided with contextual clues (eg, who they were with). Hence, this finding may reflect a limitation in our ESM template in capturing information that clients consider essential for recollection and attributing meaning to relevant data points. Therefore, future implementation studies could benefit from considering the incorporation of an open-text field at the end of an ESM questionnaire.

Despite technical usability being sufficient, clinical usability was not self-evident. Overall, a need was expressed for more personalization. Although practitioners stressed the importance of personalization, and the software provided the opportunity to adapt the content and sampling scheme, practitioners rarely used this feature. There may be 2 possible explanations for this finding. First, it may be too complex from a technical point of view because practitioners indicated that personalization was too difficult and time consuming. This aligns with prior research suggesting that future implementation must strike a balance between the need for personalization and the clinical reality of the limited time that practitioners have during clinical sessions [13,30]. Second, practitioners may not be opting for personalization because it is not straightforward to operationalize clinical questions in ESM templates [19]. This suggests that future implementation work should provide a better understanding of how personalization might help practitioners to translate specific clinical questions into ESM templates that fit clients’ individual needs. In a similar vein, the clinical usability of the data visualizations will depend on prespecified clinical applications. When the ESM is used with a clear application in mind, higher clinical utility and acceptability might be achieved [18,31]. Defining what such applications should entail is a challenging task. However, some recommendations for further implementation work can be made; for example, in a recent implementation study with individuals diagnosed with bipolar disorder, 1 suggestion made by clients and practitioners was to use the ESM for studying the effects of medication or lifestyle changes on daily life functioning [32]. Relatedly, in individuals experiencing depression, it may be worthwhile to focus on using the ESM to provide feedback relating to positive affect because this type of feedback has been suggested to benefit the reduction of depressive symptoms [33].

In contrast to recent findings [18], which found generally high compliance among patients with voice-hearing experiences (100% above the 33% cutoff), the level of compliance in our study was substantially lower (11/16, 69% above the 33% cutoff). The lower compliance in our study might be tied to the fact that the ESM setup might not have always resulted from a collaborative process between clients and practitioners (ie, limited use of personalization features). This may help explain why the ESM protocol did not always meet client expectations and was sometimes perceived as burdensome. This corroborates earlier work and stresses the importance of actively involving clients in goal setting and the setup of ESM templates [19], which will be necessary to increase patient engagement and empower clients to take an active role in their recovery process. Taken together, the findings of this ESM pilot study in specialized mental health care suggest that using a generic ESM template may be less practical because the collaborative clinical goal will determine the ESM content, schedule, and visualizations in practice.

Our findings have several implications for future research and implementation work. First, to guide further software development, more work is needed to determine how clinical goals translate into specific ESM questionnaires and sampling schemes; for example, during the initial stages of treatment, it may be more beneficial to have a detailed summary of daily life experiences to identify the patterns or contextual determinants of a client’s mental health problem (ie, the hypotheses-generating phase of diagnostic assessment). In such cases, intensive sampling for a week may be required. By contrast, there may be less need for such a dense sampling schedule when a client has been in therapy for an extended period, and the goal is to evaluate treatment and prevent relapse (ie, hypotheses-confirming evaluation and prognosis). This is also in line with recent work [12,34], which suggested that other formats of the ESM may be required for clinical use. Second, we identified a need for additional items to personalize ESM content to the needs of individual clients. Although future work could resort to items used in academic research [35], exploring co-designing items with practitioners and clients that match the experiences they want to capture outside of the therapy room may also be worthwhile. Third, although compliance is 1 indicator of burden [36], future research is needed to investigate under what circumstances the ESM is perceived as less burdensome. Fourth and last, we identified the need for other training compared with our 1-hour web-based training sessions; for instance, as suggested by the practitioners in our study and elsewhere [13], including group training with mock clients might help increase usability. However, addressing the aforementioned issues will be a prerequisite in developing practical and concrete training programs that match clinical complexity. Such training programs could be introduced into higher education programs that take a dimensional and recovery-based perspective on mental health care.

### Limitations

Several limitations should be considered when interpreting the results of this pilot study. First, and as mentioned elsewhere [37], there are numerous ways to define and measure a mobile health app’s usability, and not all of these elements were studied. Hence, other usability elements may still need to be studied (eg, mobile phone battery constraints). Second, we experienced a challenging recruitment procedure owing to the unforeseen and unique circumstances of the COVID-19 pandemic. However, participant numbers (ie, 12/142, 8.5%) exceed what usability experts consider as sufficient and recommend for pilot testing in an iterative user-centered design [38,39]. Third, our sample consists of early adopters who may not be representative of the entire population of practitioners and clients. Fourth and last, we did not implement any strategies to increase user engagement, such as gamification [40], which may be beneficial to consider in future work.

### Conclusions

In this pilot study, we designed and implemented a protocol for using an ESM template in a specialized mental health care setting. Our findings suggest that the ESM template and the software are easy to use, indicating that practitioners and clients are capable agents for using the ESM in clinical practice. Nevertheless, clients’ readiness to use (or keep using) the ESM was restricted owing to limitations in perceived usefulness. Hence, the piloted ESM protocol should not be readily implemented, and substantial adaptions are necessary. These may include providing additional sampling scheme formats, enabling personalization through codeveloped items, adding an open-text field item, and introducing a dynamic data visualization interface. To optimize the usability of ESM protocols as a mobile health assessment tool in recovery-focused psychiatry, we encourage scientists and implementation experts to focus more on collaborating with practitioners and clients in every phase of the design, evaluation, and implementation process to make meaningful translations of clinical questions into ESM templates that truly benefit and meet the specific needs of individuals.

## Appendix

Multimedia Appendix 1 Usability of the dashboard during a clinical session.

Multimedia Appendix 2 Overall usability of the dashboard.

Multimedia Appendix 3 Overall usability of the app.

Multimedia Appendix 4 Example quotes from practitioners and clients.

## Abbreviations

3G: third-generation
ESM: experience sampling method
IMPROVE: Implementing Personalized Real-Time Monitoring in Everyday Life
MAUQ: mHealth App Usability Questionnaire

## Data Availability

The data collected in this study are available from the corresponding author upon request.
