# Supplementary figures and images for: Usability of the Experience Sampling Method in Specialized Mental Health Care: Pilot Evaluation Study
Source: JMIR Form Res. 2023 Nov 21;7:e48821. doi: 10.2196/48821 (PMC10698657; doi:10.2196/48821)

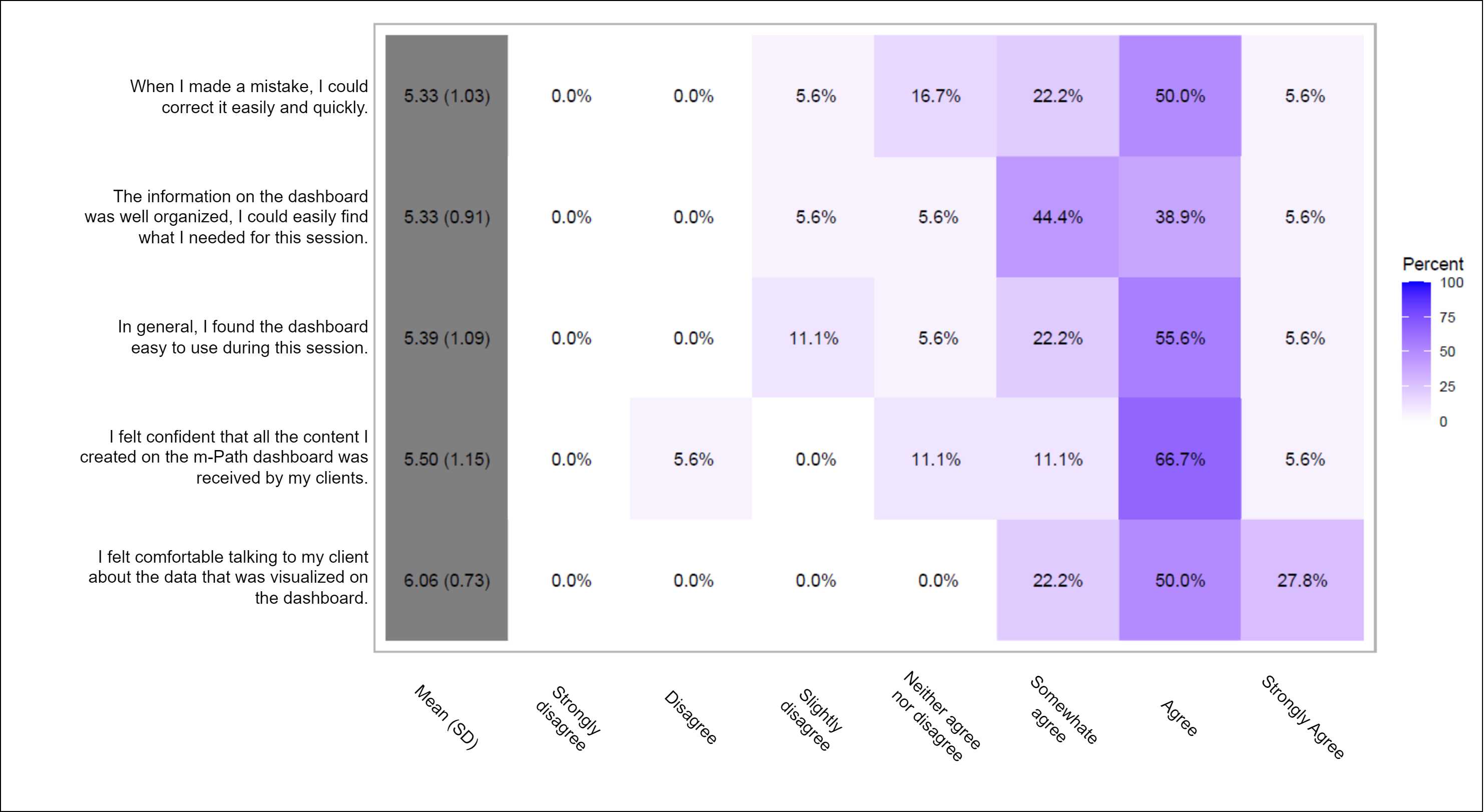

Supplement: Multimedia Appendix 1 [file formative_v7i1e48821_app1.png]

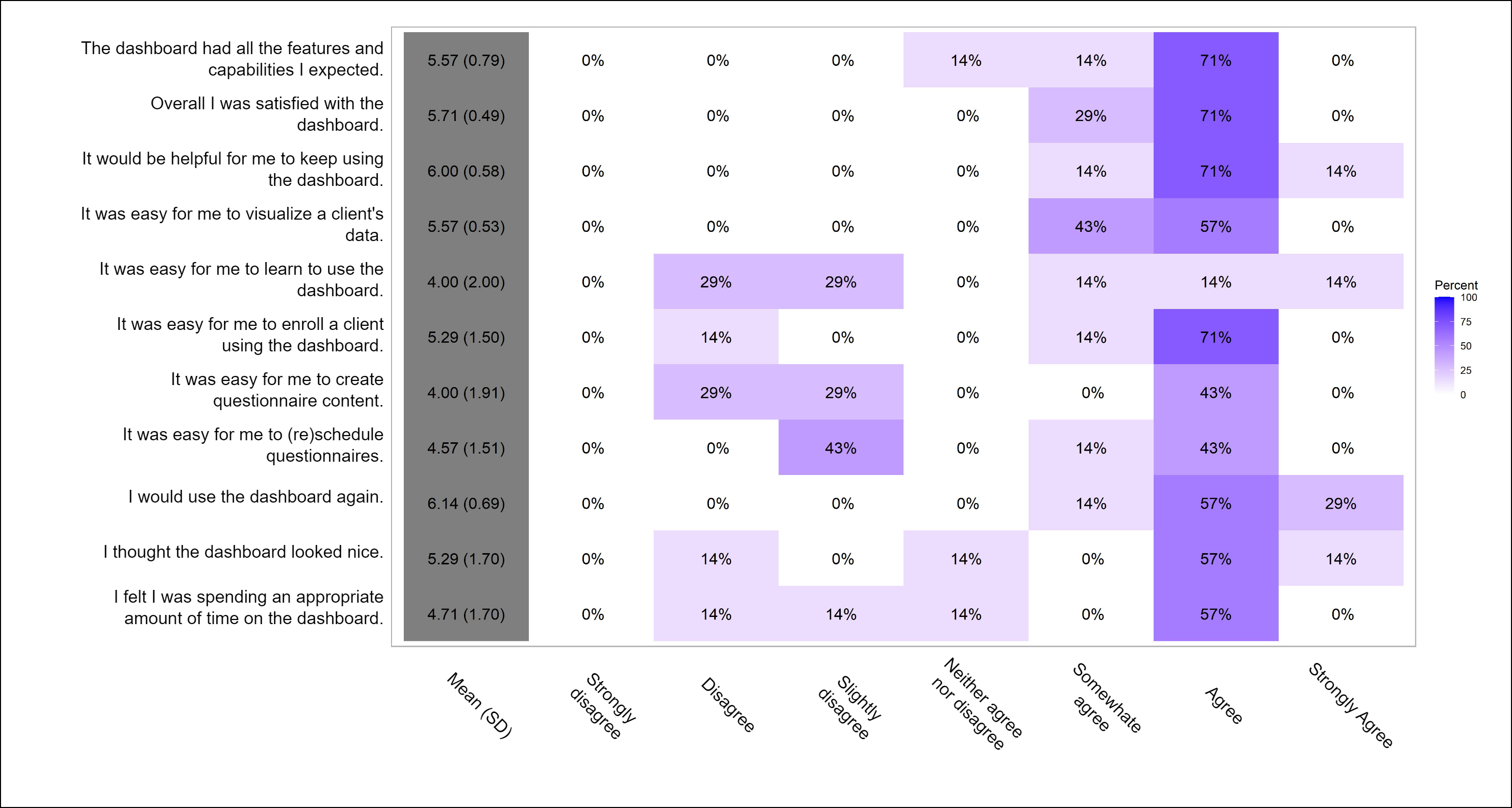

Supplement: Multimedia Appendix 2 [file formative_v7i1e48821_app2.png]

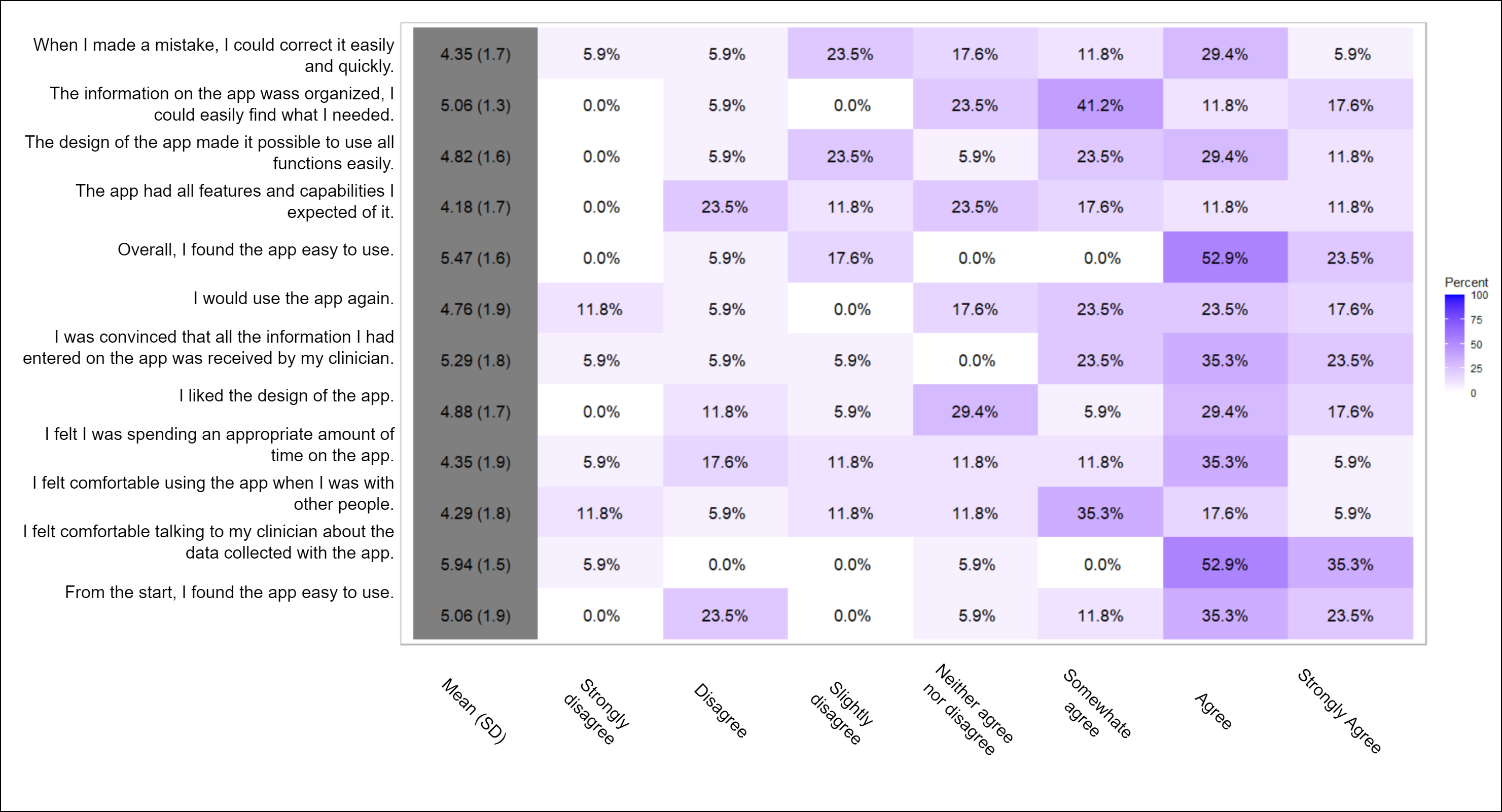

Supplement: Multimedia Appendix 3 [file formative_v7i1e48821_app3.png]
