# Supplementary material for: Usability of the Experience Sampling Method in Specialized Mental Health Care: Pilot Evaluation Study
Source: JMIR Form Res. 2023 Nov 21;7:e48821. doi: 10.2196/48821 (PMC10698657; doi:10.2196/48821)
Supplement: Multimedia Appendix 4 [file formative_v7i1e48821_app4.pdf]

Annex 4.

Selected quotes from practitioners and clients

Topic	Quote
**Using ESM**	
	Quote 1.<br>Interviewer: 'Would you like to keep using the software? Or rather not?'<br>Practitioner: 'I would like to keep using it. However, this does not mean that I will use it with every client from now on. What I mean is that I can see myself using it again and assessing whether it provides something additional, something of added value. I think it is important to remember that I have such a tool at my disposal now.'<br>*Quote 2.*<br>Practitioner: 'I asked a few clients whether they were interested in doing a week of ESM, but some indicated that they would rather not do it. I asked four people and only one wanted to try it. I think it asks quite a bit of someone to do a week of ESM and most of my clients were simply not up for that. The person that did join was enthusiastic. However, even in my recruitment, I am being selective. I had to look at whether they have a smartphone or access to the internet, and only then could I ask whether they wanted to try ESM or not.'<br>*Quote 3.*<br>Interviewer: 'How was it for you to use the app?'<br>Client: 'The first three to four days were fine. Day five was a bad day for me due to my OCD symptoms being elevated. When my phone beeped, I really thought, 'no, not again, I do not feel like it'. I also communicated this with my therapist. I think there were too many beeps in a day. My husband also commented on it. The first few days are fine, but it quickly becomes too much.'<br>*Quote 4.*<br>Interviewer: 'How was it for you to use the app?'<br>Client: 'Sometimes I found it a bit disturbing. For example, if it beeped during therapy, it felt disrespectful to my practitioner. Another example was that I got a beep during a relaxation exercise, which ruined the relaxation for me.'<br>*Quote 5.*<br>Interviewer: 'How was it for you to fill out questions on your feelings and thoughts multiple times per day?'<br>Client: 'It was a bit weird, I felt artificial. There is this feeling of needing to answer in a certain way, like I needed to show variation. But, for me, it does not really work. I cannot express how I feel 'in the moment' accurately. This might just be me, though, I just did not have the feeling I could give an accurate response to how I felt.'
**Training material**	

**Content and design**	*Quote 6.*<br>Interviewer: 'What did you think of the training we provided?'<br>Practitioner: 'I thought it was useful. However, it was online and that made it quite difficult. I was on my tablet, and it was stressful to follow both the call and do things myself. I think it is easier to follow when you are using a computer with two screens. One of my colleagues did this and it was clear to me that she was better at using the software. I think it would help, in the future, if something like this were introduced in a team meeting. Trying to follow along with only one screen made it impossible for me, I could not look at what the instructor was doing as well as try it myself.'<br>*Quote 7.*<br>Interviewer: 'What was it like to use the software in your workflow?'<br>Practitioner: 'It was easy to use, but only after I managed to install everything. I needed to get some help with the installation. I will be honest with you; I really needed the help. The manual would take too much time; if I had to do it myself, I probably would not have proceeded. Once I started using it I also asked a colleague of mine, who also joined the study, about how she interpreted collected data from my clients. I wanted to check whether we were interpreting the collected information in the same way.'<br>*Quote 8.*<br>Client: 'I found it ok to do, but I was confused by the fact that it was the same questionnaire each time.'<br><br>*Quote 9.*<br>Interviewer: 'What did you think of the add-on questions?'<br>Practitioner: 'The OCD questions, for me, were not the right type of questions. I normally ask my clients to register when they are experiencing their symptoms. In your software, the question focused more on how much burden they experience from their symptoms. I did not like the 'burden' element of it. However, I did not adjust the add-on question knowing that it was also made with input from other clinicians. Yet, for me, in the end, it was not the right type of questions to monitor OCD symptoms.'<br>*Quote 10.*<br>Interviewer: 'How was it for you to discuss the collected information with your client'<br>Practitioner: 'I showed them their data, but my clients were not very interested. I think this happened because most of the people I did this with had been with me for a long time. One week is not that meaningful to them. I think that if I started using ESM from the start of therapy, it may be more interesting. It would allow me to see change. We do something like this using patient-routine-outcome-monitoring scales and my clients also like that... so yeah, I repeat: one week says too little.'<br>*Quote 11.*<br>Interviewer: 'How was it for you to fill out questions on your feelings and thoughts multiple times per day?'<br>Client: 'It felt like it created self-awareness, which I find a good thing. You are more aware of what you feel and do. I normally do not really do that, but it is a good thing to do.'

**Personalization of content**	
	Quote 12.<br>Interviewer: 'What did you think about the option to add personalized questions?'<br>Practitioner: 'I did that for one client, and I believe it helped. I put in a question on outside activity and movement. When he got an ESM questionnaire and noticed he had been inactive, it motivated him to get outside and exercise. That was then also something you could see in the following assessment. However, it is important to note that I only added this question in dialogue with my client.'<br>*Quote 13.*<br>Interviewer: 'Did you adjust any of the content already available?'<br>Practitioner: 'Yes, for one client I added 'Partner' as an answer option on the 'With who are you?' question. I thought it was important to differentiate between being with your partner versus being with family [default option]. It was handy for determining what social situations elicit positive emotions. It was also fairly easy to add the answer option. I liked that it was possible.'<br>*Quote 14.*<br>Practitioner: 'After a while, you get faster at creating personalized content. However, I think it remains time intensive. If you work in a ward, where you see the same type of clients you can re-use your own personalized questions. However, when you have your own private practice and see a lot of different clients, I think it will be too time intensive. To implement this, I think it helps if you invest more time in it in the beginning.'<br>*Quote 15.*<br>Practitioner: 'I recall telling one of your team members that I would call him if I ran into trouble as I am bad with technology. Ultimately, I had to call as I failed to create my own questions. My client completed a week of ESM without adding my personalized questions. I expected things to go wrong and it did. However, it was probably also a bit my own fault as I am not that good with technology.'
**Data visualization**	
	Quote 16.<br>Interviewer: 'How was it for you to discuss the visualizations during a session?'<br>Practitioner: 'I generally work with a whiteboard to create visualizations of a client’s experiences, so seeing information automatically visualized is something that was rather pleasant for both my client and me. I would say things like 'on that day we see a lot of negative emotions, why was that?'. So having the visualizations was really an advantage, I can really dig deep. I generally collect similar information using a lot of homework, but seeing it automatically visualized in this way is pleasant.'<br>*Quote 17.*<br>Interviewer: 'What did you think of the visualizations, were there certain figures or graphs that you particularly liked or disliked?'<br>Practitioner: 'I thought it was well done. I have never used a pie chart [displaying time spent in context or activity] clinically, but I found it super interesting. One client also told me that it really helped her get more self-insight.'

	Quote 18.<br>Interviewer: 'What was your opinion on the data visualizations?'<br>Practitioner: 'I recall there being pie charts, bar charts, and line graphs. In the beginning, it was a bit overwhelming. You open it up and think 'What is all this?', but you can of course zoom in on those visualizations that you find relevant for that clinical session. Once you get the hang of it, I think it is quite nicely presented.'<br>*Quote 19.*<br>Interviewer: 'How was it for you to see and discuss your data with the clinician?'<br>Client: 'I found it impressive to see all the graphs. Seeing which situations I spent my time in ands how I felt in those situations. I found it fascinating.'<br>*Quote 20.*<br>Interviewer: 'Did you think the figures and graphs accurately depicted your experiences?'<br>Client: 'I think I would need a bit more experience with using it to be able to answer that question. As of now, my first impression was that it was quite difficult to understand. There is a lot of information and you simply do not know what to focus on.'<br>*Quote 21.*<br>Interviewer: 'Earlier, you mentioned that interpreting the data visualizations was difficult. Can you elaborate on this?'<br>Client: 'When the practitioner explained it, it was fine. However, I really needed that explanation. Without my practitioner, I am not sure whether I would have been able to understand it or figure out what is relevant. I think people should also not try to download this and interpret their data themselves. You really need a practitioner who can decrypt everything and helps you understand how to interpret your data and what is relevant.'
**Suggestions for improvement**	
	Quote 22.<br>Interviewer: 'What was it like to use the software in your workflow?'<br>Practitioner: 'Interesting. I do not think I could make full use of what it can do or be used for. Right now, I just tested it for a bit. However, I think a repeating assessment would also be interesting. It would allow you to explore how a client progresses through therapy. Another assessment week is something we did not yet do. I think it might be useful.'.<br>*Quote 23.*<br>Interviewer: 'Would you like to use it again in the future?'<br>Client: 'Yes, but it depends on how it is done. It was okay to do for a few days, but I would find it difficult if I had to do it longer. If I could choose, I would do it once a month for a week, but with fewer beeps a day. The content can be the same.'<br>*Quote 24.*<br>Interviewer: 'Earlier you mentioned additional training is necessary. Do you have something in mind?'

	Practitioner: 'Maybe some training using example cases? So, more from the perspective of how I can use it in therapy. The training that I received went a bit too fast. Our sector is not familiar with the use of digital tools, and a lot of people in our sector are not good with technology. I include myself in this list. I would have liked to get more support. What we do with interns is we provide them with a trial period before we allow them to try something with a client, I think something like that would be nice. If you feel like you are inexperienced with the software, it is also not nice to use it in practice. So, practice sessions in a team would be important before using it with clients.'<br>*Quote 25.*<br>Client: 'For me the questions could be a bit more descriptive. Perhaps you could use open questions? I do understand that this makes it more difficult to compare responses in nice graphs and patterns.'<br>*Quote 26.*<br>Client: 'I would suggest further differentiating answer options to some questions. I had moments when I was with people, like my music teacher, whom I could not put in one of the categories. What would also help here is maybe just an answer option where I could fill out who I am with in an open text box.'<br>*Quote 27.*<br>Practitioner: 'As with face-to-face therapy, I would find it pleasant to have a digital notebook to make annotations as we go over the data.'<br>*Quote 28.*<br>Interviewer: 'Is there something you would like to change to the app?'<br>Client: 'I would put a bit more color into the questionnaire. It feels a bit monotone, sorry.'<br>*Quote 29.*<br>Interviewer: 'What did you think of the questionnaires you received multiple times per day? Were the questions relevant for you?'<br>Client: 'I think the questions were ok. However, after a while, you get so used to the questionnaire that you fill it out without properly reading the questions. I think it then happened that I sometimes filled it out wrong. For example, I sometimes thought the question said compulsive thoughts as opposed to compulsive behaviors and then gave a wrong answer. Perhaps it would be an idea to underline or highlight certain parts of the question?'

*Quotes have been translated from Dutch and paraphrased to improve comprehension
